# Supplementary material for: Metabolic drug survey highlights cancer cell dependencies and vulnerabilities
Source: Nat Commun. 2021 Dec 14;12:7190. doi: 10.1038/s41467-021-27329-x (PMC8671470; doi:10.1038/s41467-021-27329-x)
Supplement: Supplementary file 1 — Supplementary Information [file 41467_2021_27329_MOESM1_ESM.pdf]

SUPPLEMENTARY INFORMATION

Metabolic drug survey highlights cancer cell dependencies and vulnerabilities

T. Pemovska, J. W. Bigenzahn, I. Srndic, A. Lercher, A. Bergthaler, A. César-

Razquin, F. Kartnig, C. Kornauth, P. Valent, P. B. Staber, G. Superti-Furga.

Content of Supplementary Information:

Supplementary Figure 1: Distribution of metabolic inhibitors affecting cellular viability per metabolic pathway / process and identification of selectively acting compounds in disease-specific manner.

Supplementary Figure 2: Metabolic drug sensitivity testing classifies myeloid leukemia cell lines in 5 functional groups.

Supplementary Figure 3: Metabolic phenotypes of the myeloid leukemia cell lines.

Supplementary Figure 4: Analysis of publicly available targeted metabolomics data from the CCLE Resource.

Supplementary Figure 5: Significant genotype to phenotype associations.

Supplementary Figure 6: 3D drug synergy visualizations for 11 different drug-drug interactions in HL-60, Mono-Mac-6, BV-173, and MV4-11 cells.

Supplementary Figure 7: HL-60 cells are vulnerable to PI3K inhibition.

Supplementary Figure 8: HL-60 cells are dependent on de novo FA synthesis.

Supplementary Figure 9: *SLC16A1* essentiality and determinants of sensitivity to the SLC16A1 inhibitor AZD3965.

Supplementary Table 1: Patients' characteristics.

Supplementary References

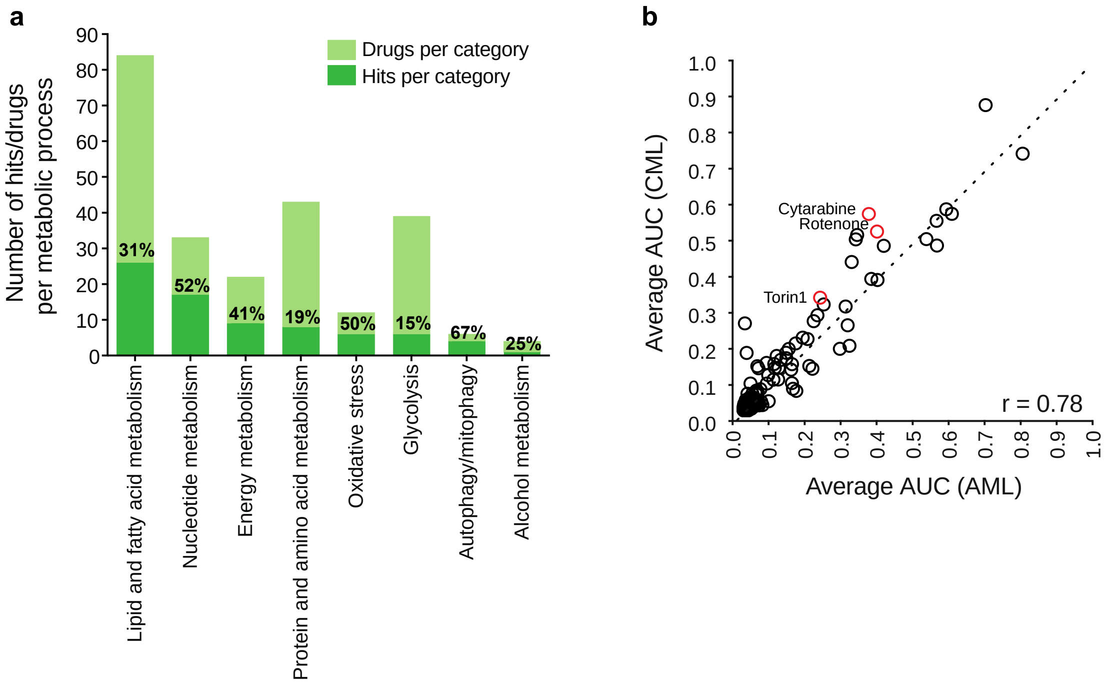

Supplementary Fig. 1| Distribution of metabolic inhibitors affecting cellular viability per metabolic pathway / process and identification of selectively acting compounds in disease-specific manner. a, Metabolic pathway coverage of compounds that had an effect on cell viability in the tested cell lines highlighting the percentage of hits in relation of all compounds in a given metabolic category. **b**, Correlation of the average metabolic drug sensitivity profile of AML and CML cell lines showing disease specific vulnerabilities.

a

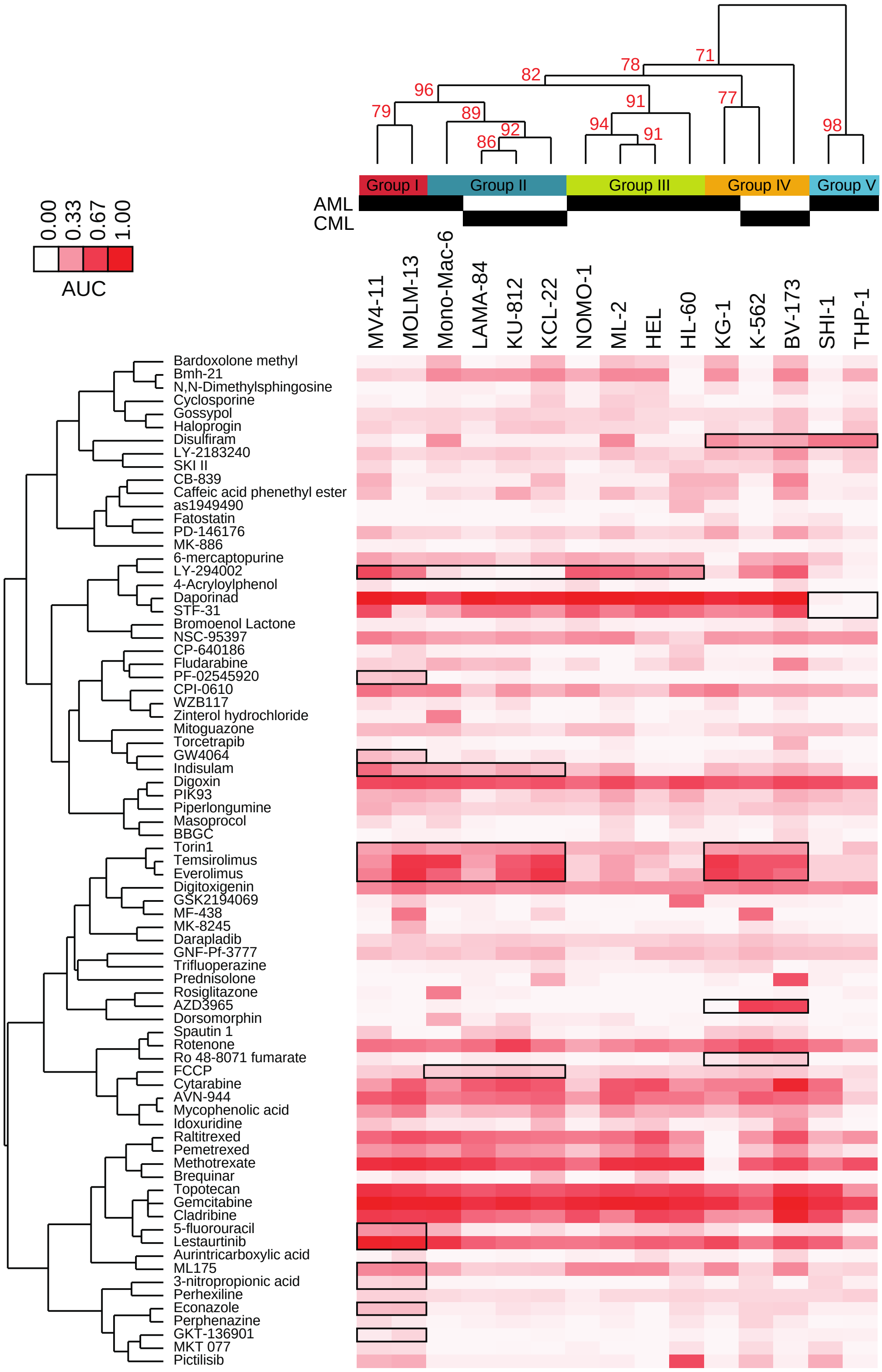

79
96
89
92
86
82
78
71
91
94
91
77
98
Group I
Group II
Group III
Group IV
Group V
AML
CML
0.00
0.33
0.67
1.00
AUC
MV4-11
MOLM-13
Mono-Mac-6
LAMA-84
KU-812
KCL-22
NOMO-1
ML-2
HEL
HL-60
KG-1
K-562
BV-173
SHI-1
THP-1
Bardoxolone methyl
Bmh-21
N,N-Dimethylsphingosine
Cyclosporine
Gossypol
Haloprogin
Disulfiram
LY-2183240
SKI II
CB-839
Caffeic acid phenethyl ester
as1949490
Fatostatin
PD-146176
MK-886
6-mercaptopurine
LY-294002
4-Acryloylphenol
Daporinad
STF-31
Bromoenol Lactone
NSC-95397
CP-640186
Fludarabine
PF-02545920
CPI-0610
WZB117
Zinterol hydrochloride
Mitoguazone
Torcetrapib
GW4064
Indisulam
Digoxin
PIK93
Piperlongumine
Masoprocol
BBGC
Torin1
Temsirolimus
Everolimus
Digitoxigenin
GSK2194069
MF-438
MK-8245
Darapladib
GNF-Pf-3777
Trifluoperazine
Prednisolone
Rosiglitazone
AZD3965
Dorsomorphin
Spautin 1
Rotenone
Ro 48-8071 fumarate
FCCP
Cytarabine
AVN-944
Mycophenolic acid
Idoxuridine
Raltitrexed
Pemetrexed
Methotrexate
Brequinar
Topotecan
Gemcitabine
Cladribine
5-fluorouracil
Lestaurtinib
Aurintricarboxylic acid
ML175
3-nitropropionic acid
Perhexiline
Econazole
Perphenazine
GKT-136901
MKT 077
Pictilisib

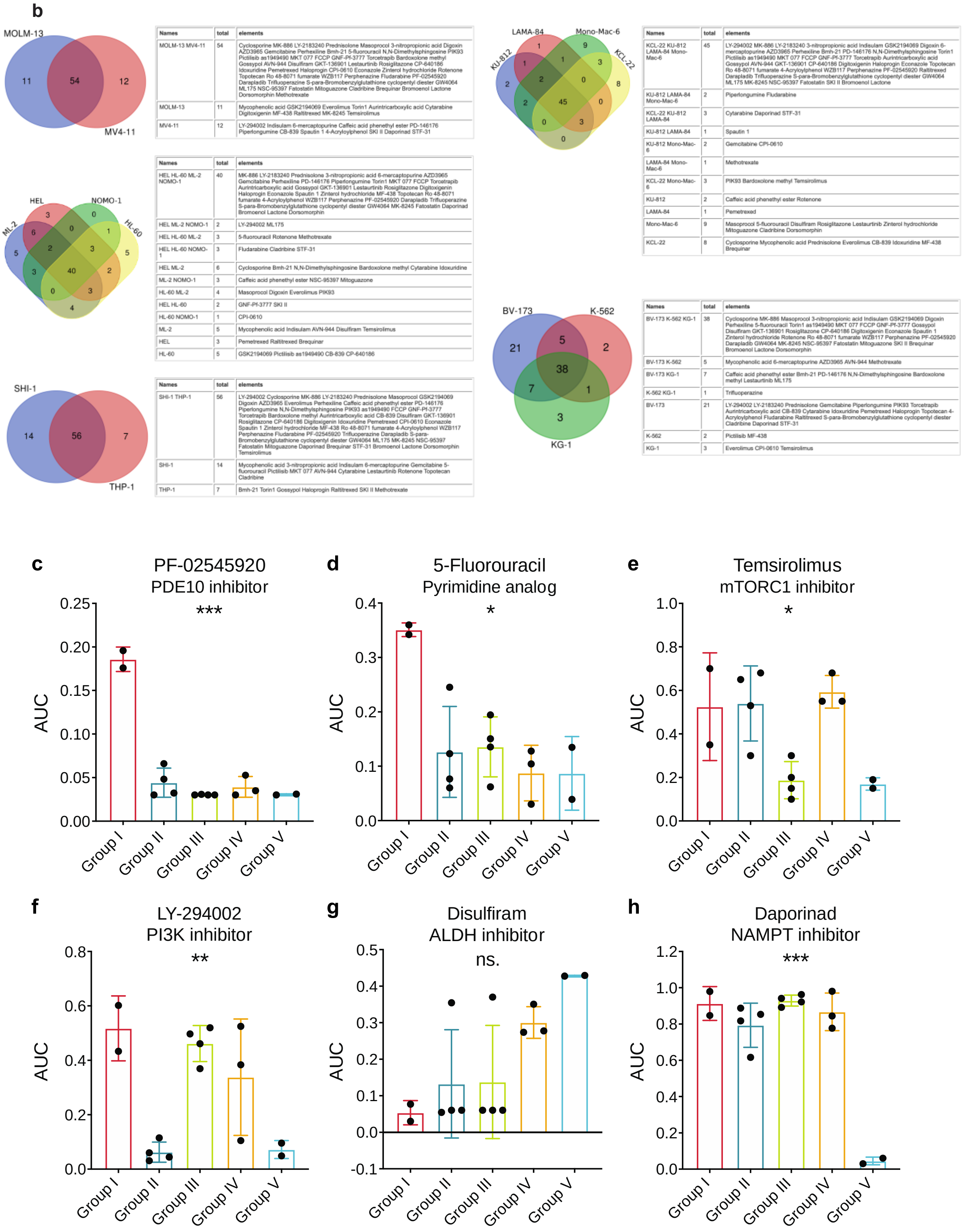

Names	total	elements
MOLM-13 MV4-11	54	Cyclosporine MK-886 LY-2183240 Prednisolone Masoprocol 3-nitropropionic acid Digoxin AZD3965 Gemcitabine Perhexiline Bmh-21 5-fluorouracil N,N-Dimethylsphingosine PIK93 Pictilisib as1949490 MKT 077 FCCP GNF-Pf-3777 Torcetrapib Bardoxolone methyl Gossypol AVN-944 Disulfiram GKT-136901 Lestaurtinib Rosiglitazone CP-640186 Idoxuridine Pemetrexed Haloprogin CPI-0610 Econazole Zinterol hydrochloride Rotenone Topotecan Ro 48-8071 fumarate WZB117 Perphenazine Fludarabine PF-02545920 Darapladib Trifluoperazine S-para-Bromobenzylglutathione cyclopentyl diester GW4064 ML175 NSC-95397 Fatostatin Mitoguazone Cladribine Brequinar Bromoenol Lactone Dorsomorphin Methotrexate
MOLM-13	11	Mycophenolic acid GSK2194069 Everolimus Torin1 Aurintricarboxylic acid Cytarabine Digitoxigenin MF-438 Raltitrexed MK-8245 Temsirolimus
MV4-11	12	LY-294002 Indisulam 6-mercaptopurine Caffeic acid phenethyl ester PD-146176 Piperlongumine CB-839 Spautin 1 4-Acryloylphenol SKI II Daporinad STF-31

Names	total	elements
HEL HL-60 ML-2 NOMO-1	40	MK-886 LY-2183240 Prednisolone 3-nitropropionic acid 6-mercaptopurine AZD3965 Gemcitabine Perhexiline PD-146176 Piperlongumine Torin1 MKT 077 FCCP Torcetrapib Aurintricarboxylic acid Gossypol GKT-136901 Lestaurtinib Rosiglitazone Digitoxigenin Haloprogin Econazole Spautin 1 Zinterol hydrochloride MF-438 Topotecan Ro 48-8071 fumarate 4-Acryloylphenol WZB117 Perphenazine PF-02545920 Darapladib Trifluoperazine S-para-Bromobenzylglutathione cyclopentyl diester GW4064 MK-8245 Fatostatin Daporinad Bromoenol Lactone Dorsomorphin
HEL ML-2 NOMO-1	2	LY-294002 ML175
HEL HL-60 ML-2	3	5-fluorouracil Rotenone Methotrexate
HEL HL-60 NOMO-1	3	Fludarabine Cladribine STF-31
HEL ML-2	6	Cyclosporine Bmh-21 N,N-Dimethylsphingosine Bardoxolone methyl Cytarabine Idoxuridine
ML-2 NOMO-1	3	Caffeic acid phenethyl ester NSC-95397 Mitoguazone
HL-60 ML-2	4	Masoprocol Digoxin Everolimus PIK93
HEL HL-60	2	GNF-Pf-3777 SKI II
HL-60 NOMO-1	1	CPI-0610
ML-2	5	Mycophenolic acid Indisulam AVN-944 Disulfiram Temsirolimus
HEL	3	Pemetrexed Raltitrexed Brequinar
HL-60	5	GSK2194069 Pictilisib as1949490 CB-839 CP-640186

Names	total	elements
SHI-1 THP-1	56	LY-294002 Cyclosporine MK-886 LY-2183240 Prednisolone Masoprocol GSK2194069 Digoxin AZD3965 Everolimus Perhexiline Caffeic acid phenethyl ester PD-146176 Piperlongumine N,N-Dimethylsphingosine PIK93 as1949490 FCCP GNF-Pf-3777 Torcetrapib Bardoxolone methyl Aurintricarboxylic acid CB-839 Disulfiram GKT-136901 Rosiglitazone CP-640186 Digitoxigenin Idoxuridine Pemetrexed CPI-0610 Econazole Spautin 1 Zinterol hydrochloride MF-438 Ro 48-8071 fumarate 4-Acryloylphenol WZB117 Perphenazine Fludarabine PF-02545920 Trifluoperazine Darapladib S-para-Bromobenzylglutathione cyclopentyl diester GW4064 ML175 MK-8245 NSC-95397 Fatostatin Mitoguazone Daporinad Brequinar STF-31 Bromoenol Lactone Dorsomorphin Temsirolimus
SHI-1	14	Mycophenolic acid 3-nitropropionic acid Indisulam 6-mercaptopurine Gemcitabine 5-fluorouracil Pictilisib MKT 077 AVN-944 Cytarabine Lestaurtinib Rotenone Topotecan Cladribine
THP-1	7	Bmh-21 Torin1 Gossypol Haloprogin Raltitrexed SKI II Methotrexate

Names	total	elements
KCL-22 KU-812 LAMA-84 Mono-Mac-6	45	LY-294002 MK-886 LY-2183240 3-nitropropionic acid Indisulam GSK2194069 Digoxin 6-mercaptopurine AZD3965 Perhexiline Bmh-21 PD-146176 N,N-Dimethylsphingosine Torin1 Pictilisib as1949490 MKT 077 FCCP GNF-Pf-3777 Torcetrapib Aurintricarboxylic acid Gossypol AVN-944 GKT-136901 CP-640186 Digitoxigenin Haloprogin Econazole Topotecan Ro 48-8071 fumarate 4-Acryloylphenol WZB117 Perphenazine PF-02545920 Raltitrexed Darapladib Trifluoperazine S-para-Bromobenzylglutathione cyclopentyl diester GW4064 ML175 MK-8245 NSC-95397 Fatostatin SKI II Bromoenol Lactone
KU-812 LAMA-84 Mono-Mac-6	2	Piperlongumine Fludarabine
KCL-22 KU-812 LAMA-84	3	Cytarabine Daporinad STF-31
KU-812 LAMA-84	1	Spautin 1
KU-812 Mono-Mac-6	2	Gemcitabine CPI-0610
LAMA-84 Mono-Mac-6	1	Methotrexate
KCL-22 Mono-Mac-6	3	PIK93 Bardoxolone methyl Temsirolimus
KU-812	2	Caffeic acid phenethyl ester Rotenone
LAMA-84	1	Pemetrexed
Mono-Mac-6	9	Masoprocol 5-fluorouracil Disulfiram Rosiglitazone Lestaurtinib Zinterol hydrochloride Mitoguazone Cladribine Dorsomorphin
KCL-22	8	Cyclosporine Mycophenolic acid Prednisolone Everolimus CB-839 Idoxuridine MF-438 Brequinar

Names	total	elements
BV-173 K-562 KG-1	38	Cyclosporine MK-886 Masoprocol 3-nitropropionic acid Indisulam GSK2194069 Digoxin Perhexiline 5-fluorouracil Torin1 as1949490 MKT 077 FCCP GNF-Pf-3777 Gossypol Disulfiram GKT-136901 Rosiglitazone CP-640186 Digitoxigenin Econazole Spautin 1 Zinterol hydrochloride Rotenone Ro 48-8071 fumarate WZB117 Perphenazine PF-02545920 Darapladib GW4064 MK-8245 NSC-95397 Fatostatin Mitoguazone SKI II Brequinar Bromoenol Lactone Dorsomorphin
BV-173 K-562	5	Mycophenolic acid 6-mercaptopurine AZD3965 AVN-944 Methotrexate
BV-173 KG-1	7	Caffeic acid phenethyl ester Bmh-21 PD-146176 N,N-Dimethylsphingosine Bardoxolone methyl Lestaurtinib ML175
K-562 KG-1	1	Trifluoperazine
BV-173	21	LY-294002 LY-2183240 Prednisolone Gemcitabine Piperlongumine PIK93 Torcetrapib Aurintricarboxylic acid CB-839 Cytarabine Idoxuridine Pemetrexed Haloprogin Topotecan 4-Acryloylphenol Fludarabine Raltitrexed S-para-Bromobenzylglutathione cyclopentyl diester Cladribine Daporinad STF-31
K-562	2	Pictilisib MF-438
KG-1	3	Everolimus CPI-0610 Temsirolimus

Supplementary Fig. 2| Metabolic drug sensitivity testing classifies myeloid leukemia cell lines in 5 functional groups. a, Unsupervised hierarchical clustering of the 77 viability affecting drugs is presented as a heatmap across 15 cell lines.

Clustering was performed using the complete linkage clustering method and Euclidian and Spearman distance measures of the AUC values of cell lines and drugs, respectively. The response profiles of several compounds and compounds classes display differential effects across the cell lines tested and are outlined with black boxes. The reproducibility of the clusters was evaluated by randomly resampling 10,000 bootstrap samples of the original dataset and calculating the frequency at which each cluster appears in the hierarchical clustering of the bootstrap replicates. The numbers in the dendrogram tree of the cell lines indicate the approximately unbiased (AU) empirical frequencies (0-100%) from the multiscale bootstrap resampling implemented in the Pvclust R-package. **b**, Venn diagrams depicting concordant and discordant drug responses within the functional taxonomic clusters defined in Fig. 2c. **c-h**, Bar graphs depicting the distribution of AUC values for selected compounds significantly contributing to the grouping of cell lines (Group I, n=2; Group II, n=4; Group III, n=4; Group IV, n=3; and Group V, n=2) shown in **Fig. 2** (an ordinary one-way ANOVA analysis; FDR of 10% was deemed significant; * Padj ≤ 0.1; ** Padj ≤ 0.05; *** Padj ≤ 0.01; ns not significant). Error bars indicate mean ± SD.

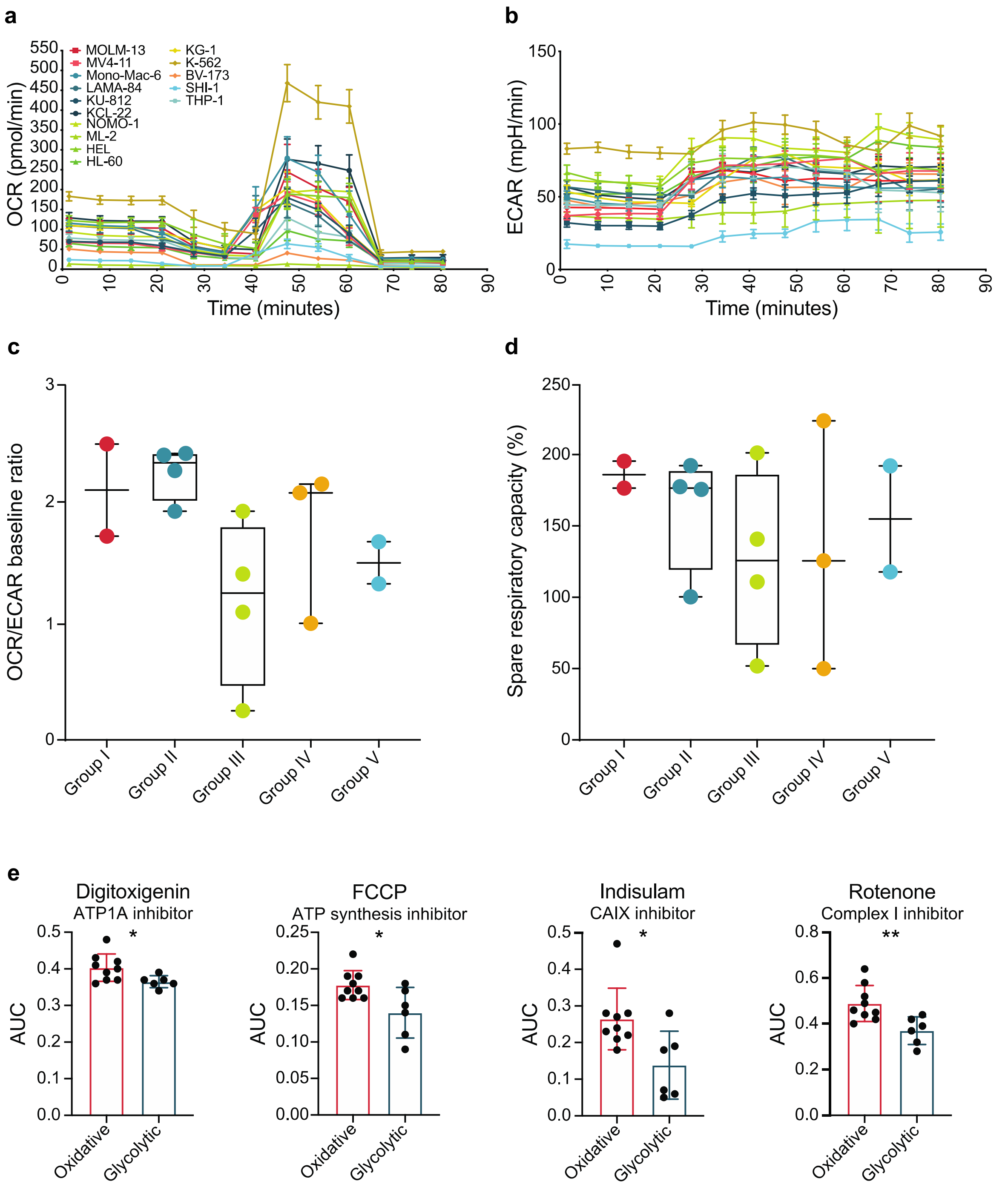

Supplementary Fig. 3| Metabolic phenotypes of the myeloid leukemia cell lines. a-b, Measurement of oxygen consumption rate (OCR) and extracellular acidification rate (ECAR) with the Seahorse analyzer XF96 at basal level and after consecutive injections of oligomycin (1μM), FCCP (1μM), and antimycin A (1μM) / rotenone (1μM) (n = 8 technical replicates; error bars mean ± SD). **c-d**, Box and whiskers (min to max) plot showing the basal bioenergetics state (the mean OCR at baseline (n = 8 technical

replicates) divided by the mean ECAR at baseline (n = 8 technical replicates) for each individual cell line (n=15) from the second baseline timepoint at approximately 8 minutes after assay start) and percentage spare respiratory capacity of the myeloid cell lines. The black line in the middle of the box depicts the median and the box extends from the 25th to 75th percentiles. **e**, Bar graph illustrating a significant difference in sensitivity to inhibitors targeting energy metabolism between more oxidative cell lines (ones falling in Group I, II and IV; n=9) and the more glycolytic cell lines (ones in Group III and V; n=6). Data is presented as mean ± SD; Unpaired two-tailed T-test; * $P < 0.05$, ** $P < 0.01$ (digitoxigenin $P = 0.0358$; FCCP $P = 0.0182$; indisulam $P = 0.0272$; rotenone $P = 0.0081$).

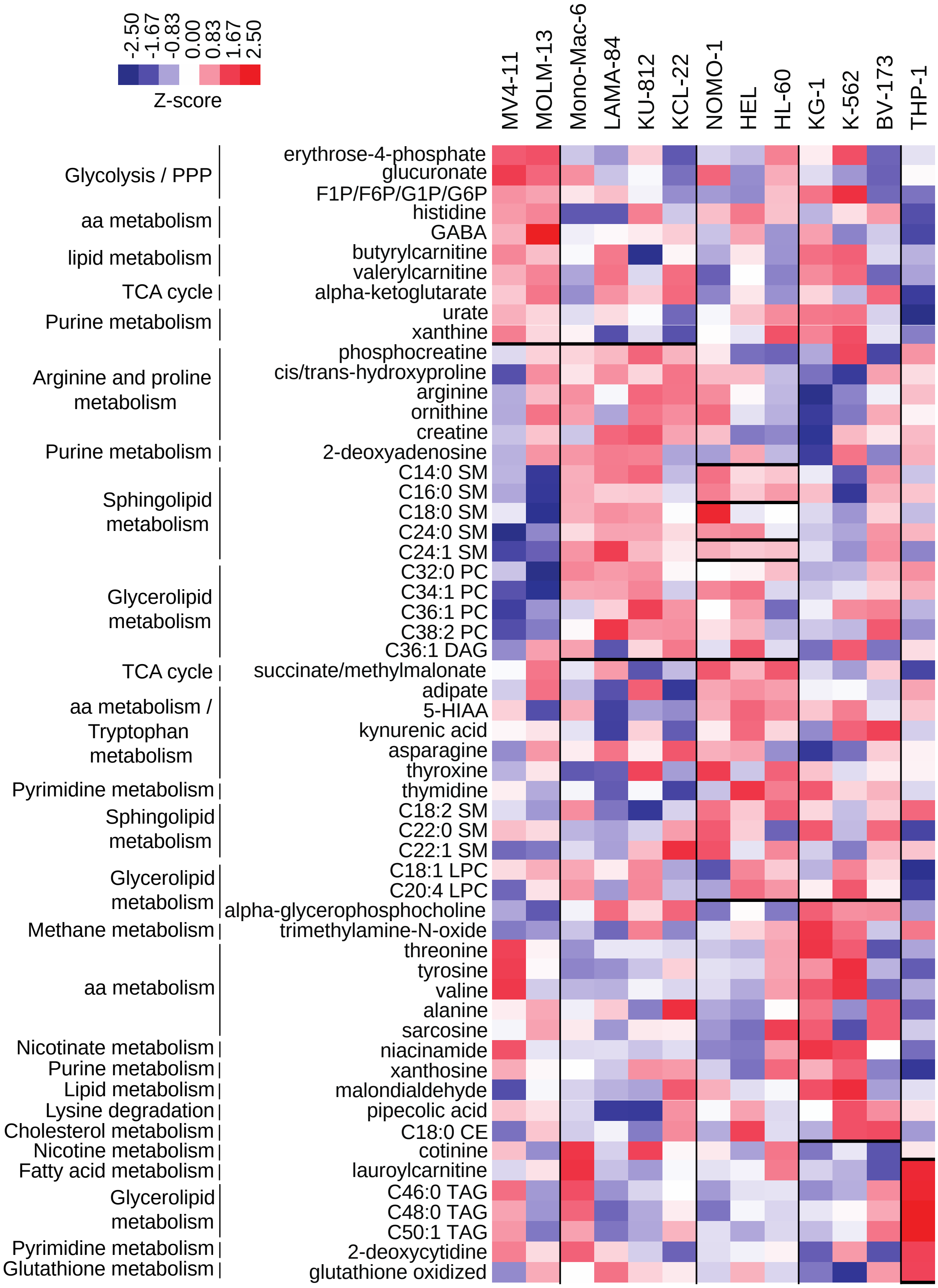

Supplementary Fig. 4| Analysis of publicly available targeted metabolomics data from the CCLE Resource[1]. The levels of 57 metabolites, represented as Z-scores and visualized with a heatmap, were significantly different between at least two of the drug sensitivity pre-defined groups with a two-way ANOVA analysis (FDR of 10% was

deemed significant). For analysis the clean imputed Log2 transformed values provided in the original publication were used.

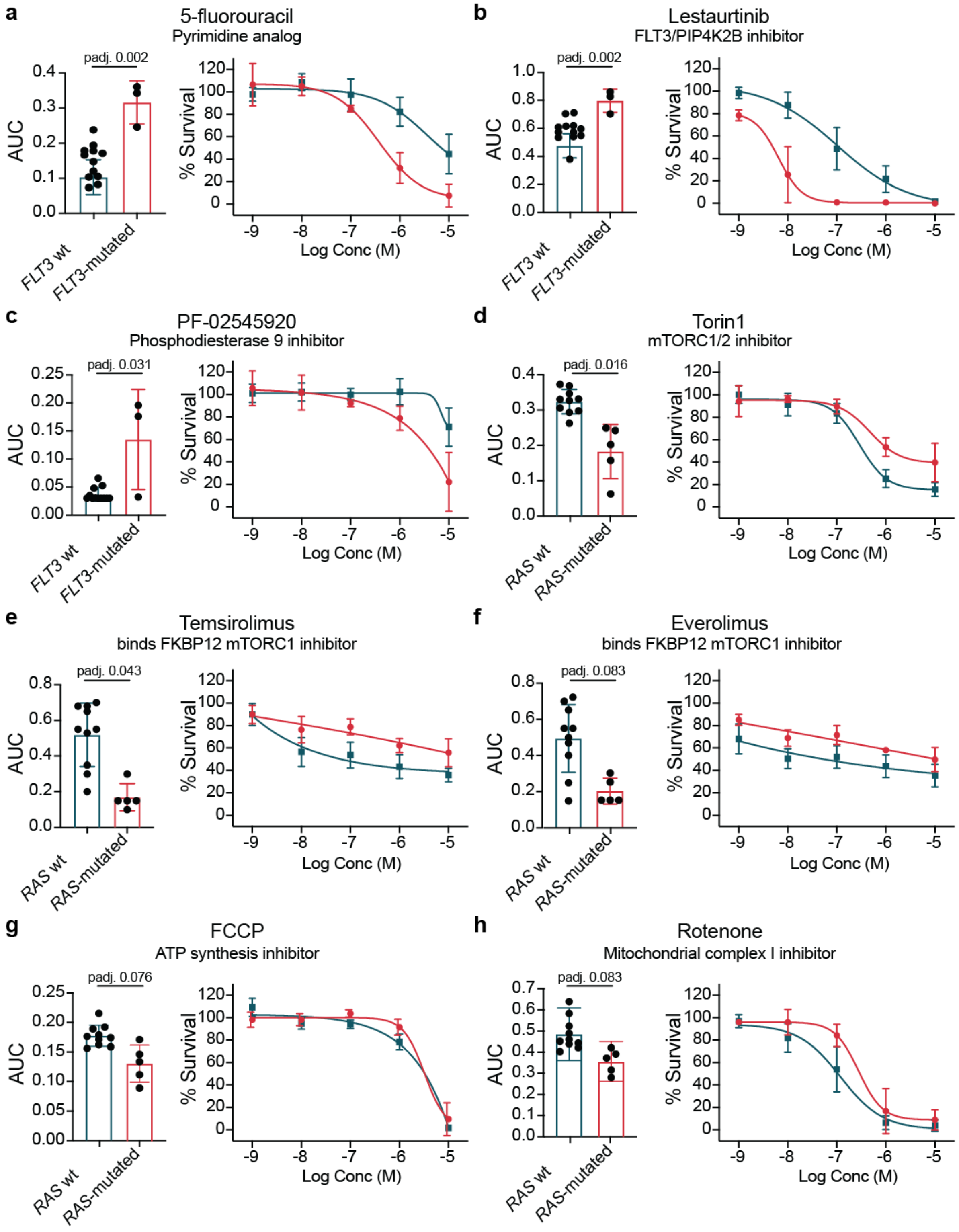
a
5-fluorouracil
Pyrimidine analog
padj. 0.002
AUC
FLT3 wt
FLT3-mutated
% Survival
Log Conc (M)
b
Lestaurtinib
FLT3/PIP4K2B inhibitor
padj. 0.002
c
PF-02545920
Phosphodiesterase 9 inhibitor
padj. 0.031
d
Torin1
mTORC1/2 inhibitor
padj. 0.016
RAS wt
RAS-mutated
e
Temsirolimus
binds FKBP12 mTORC1 inhibitor
padj. 0.043
f
Everolimus
binds FKBP12 mTORC1 inhibitor
padj. 0.083
g
FCCP
ATP synthesis inhibitor
padj. 0.076
h
Rotenone
Mitochondrial complex I inhibitor
padj. 0.083

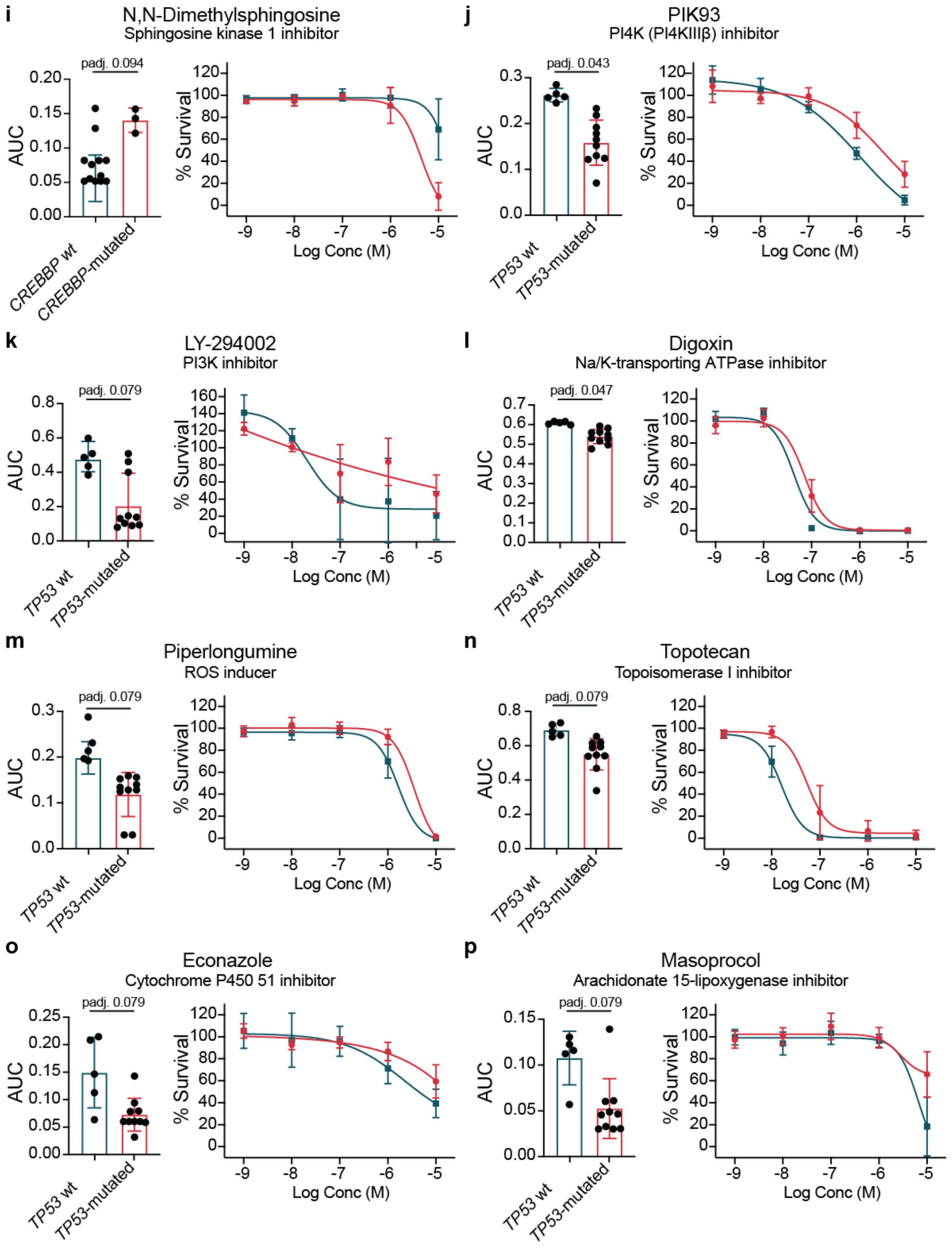

Supplementary Fig. 5| Significant genotype to phenotype associations. a-p, Box-plots and mean dose response curves depicting sensitivity of several metabolic modifiers that have significantly higher or lower sensitivity in *FLT3-ITD* (**a-c**), *RAS* (**d-**

h), *CREBBP* (**i**), and *TP53* (**j-p**) mutant versus wild-type cell lines as depicted in **Fig. 3a**. Error bars signify mean ± SD and the difference in response was assessed with a two-tailed unpaired *T*-test after multiple testing correction (FDR of 10% was deemed significant; * Padj ≤ 0.1; ** Padj < 0.05; *** Padj < 0.01).

HL-60

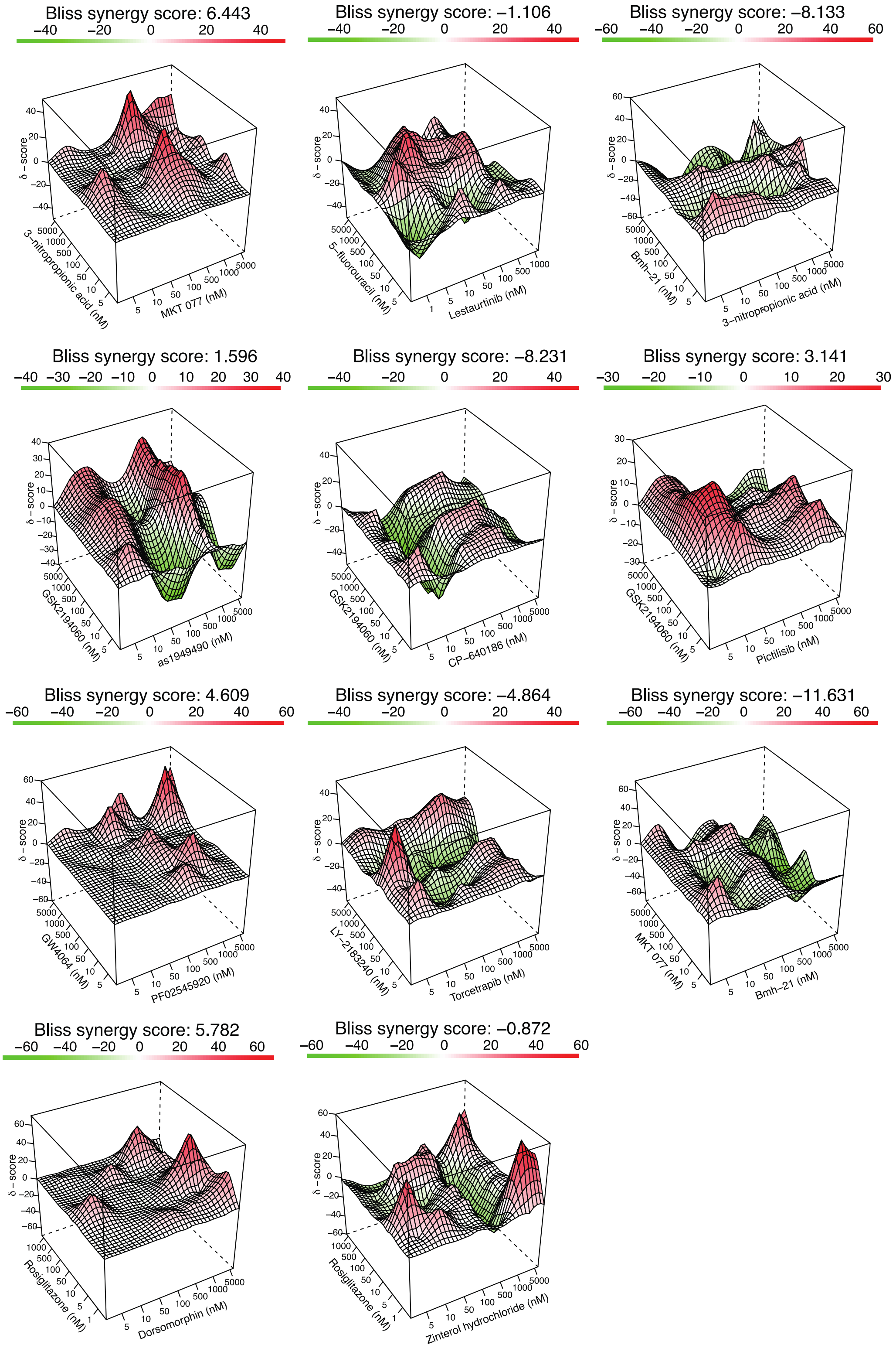
Bliss synergy score: 6.443
δ − score
3−nitropropionic acid (nM)
MKT 077 (nM)
Bliss synergy score: −1.106
δ − score
5−fluorouracil (nM)
Lestaurtinib (nM)
Bliss synergy score: −8.133
δ − score
Bmh−21 (nM)
3−nitropropionic acid (nM)
Bliss synergy score: 1.596
δ − score
GSK2194060 (nM)
as1949490 (nM)
Bliss synergy score: −8.231
δ − score
GSK2194060 (nM)
CP−640186 (nM)
Bliss synergy score: 3.141
δ − score
GSK2194060 (nM)
Pictilisib (nM)
Bliss synergy score: 4.609
δ − score
GW4064 (nM)
PF02545920 (nM)
Bliss synergy score: −4.864
δ − score
LY−2183240 (nM)
Torcetrapib (nM)
Bliss synergy score: −11.631
δ − score
MKT 077 (nM)
Bmh−21 (nM)
Bliss synergy score: 5.782
δ − score
Rosiglitazone (nM)
Dorsomorphin (nM)
Bliss synergy score: −0.872
δ − score
Rosiglitazone (nM)
Zinterol hydrochloride (nM)

Mono-Mac-6

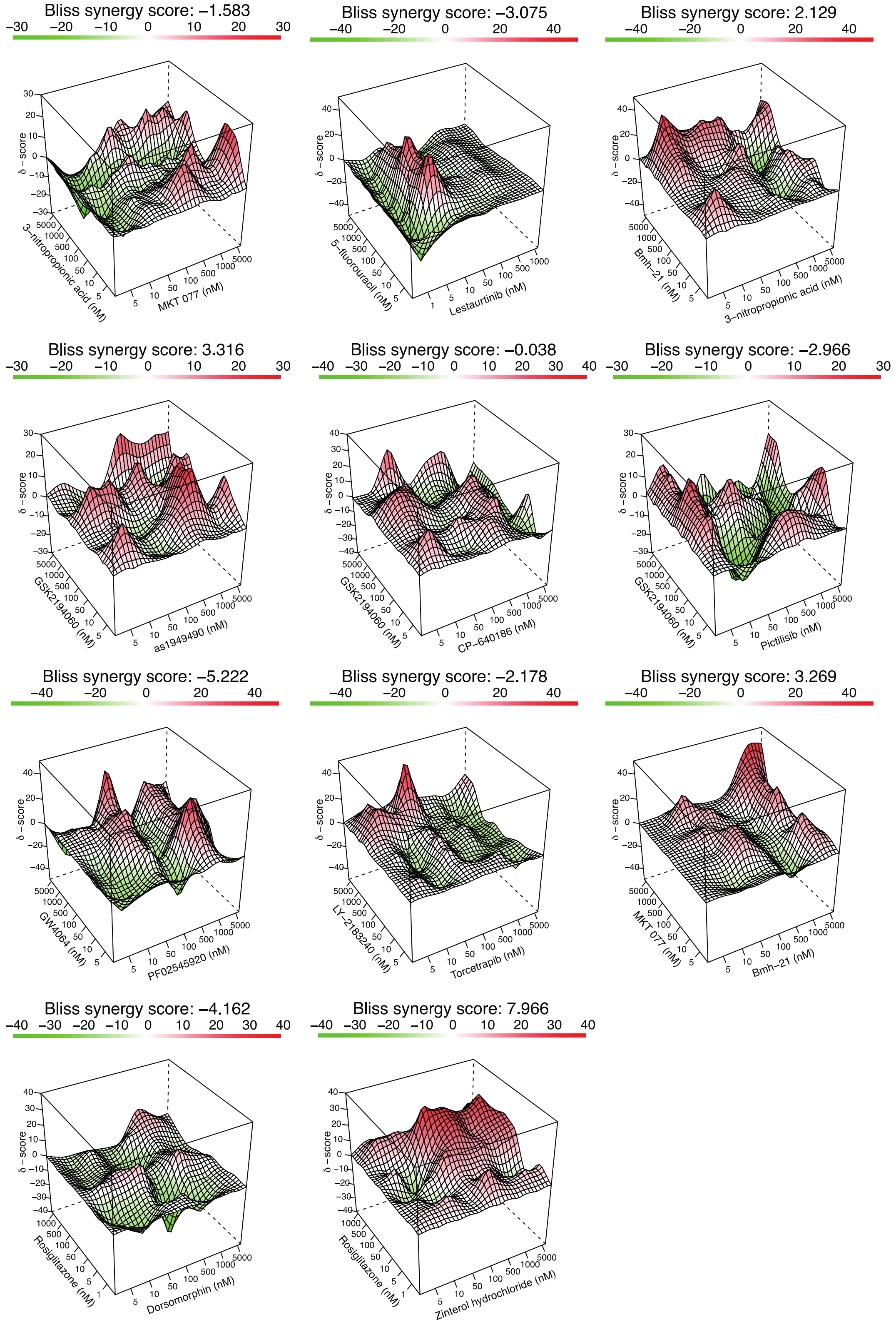
Bliss synergy score: −1.583
δ − score
3−nitropropionic acid (nM)
MKT 077 (nM)
Bliss synergy score: −3.075
δ − score
5−fluorouracil (nM)
Lestaurtinib (nM)
Bliss synergy score: 2.129
δ − score
Bmh−21 (nM)
3−nitropropionic acid (nM)
Bliss synergy score: 3.316
δ − score
GSK2194060 (nM)
as1949490 (nM)
Bliss synergy score: −0.038
δ − score
GSK2194060 (nM)
CP−640186 (nM)
Bliss synergy score: −2.966
δ − score
GSK2194060 (nM)
Pictilisib (nM)
Bliss synergy score: −5.222
δ − score
GW4064 (nM)
PF02545920 (nM)
Bliss synergy score: −2.178
δ − score
LY−2183240 (nM)
Torcetrapib (nM)
Bliss synergy score: 3.269
δ − score
MKT 077 (nM)
Bmh−21 (nM)
Bliss synergy score: −4.162
δ − score
Rosiglitazone (nM)
Dorsomorphin (nM)
Bliss synergy score: 7.966
δ − score
Rosiglitazone (nM)
Zinterol hydrochloride (nM)

BV-173

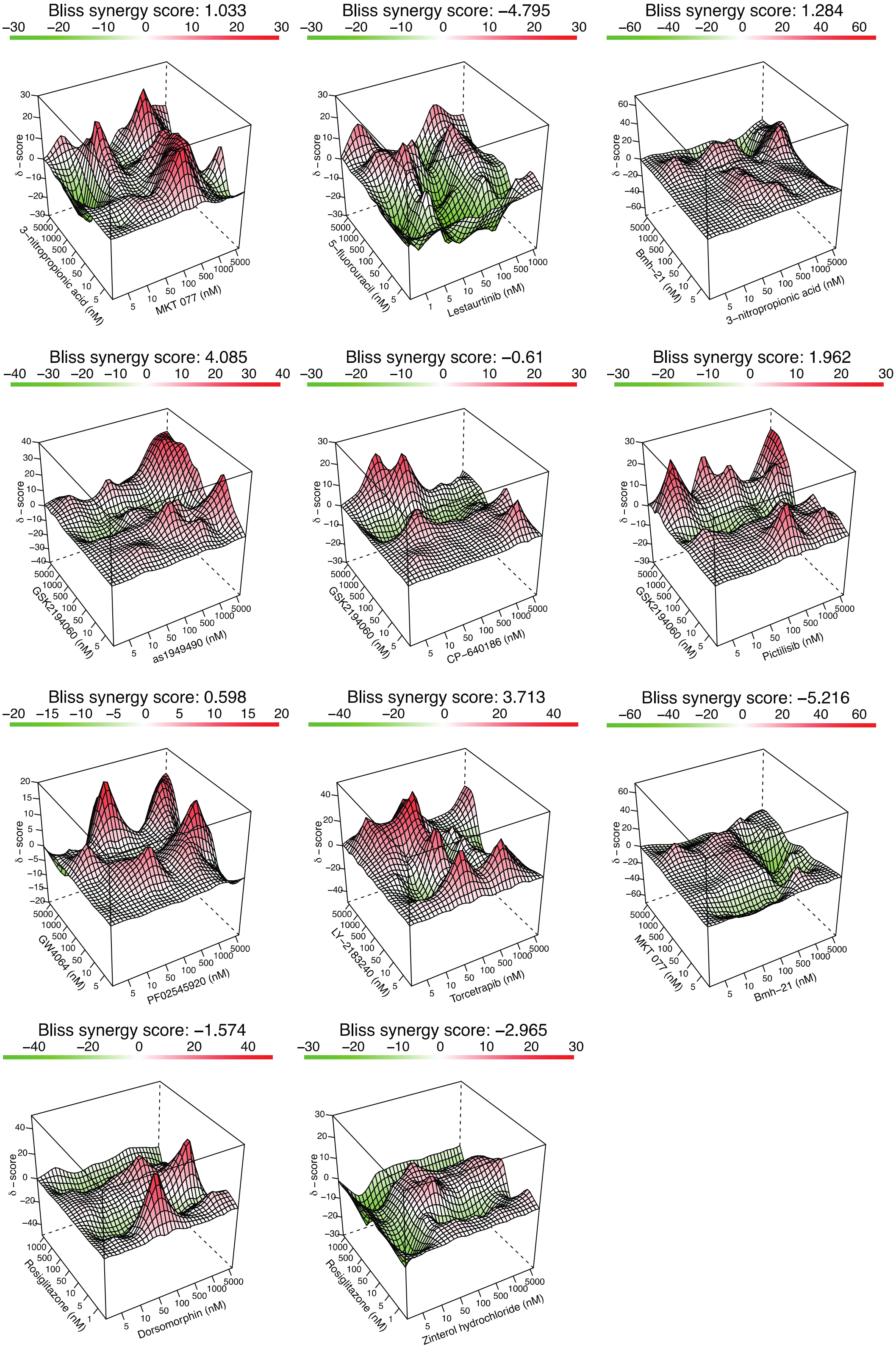

Bliss synergy score: 1.033
δ − score
3−nitropionic acid (nM)
MKT 077 (nM)
Bliss synergy score: −4.795
5−fluorouracil (nM)
Lestaurtinib (nM)
Bliss synergy score: 1.284
Bmh−21 (nM)
3−nitropropionic acid (nM)
Bliss synergy score: 4.085
GSK2194060 (nM)
as1949490 (nM)
Bliss synergy score: −0.61
GSK2194060 (nM)
CP−640186 (nM)
Bliss synergy score: 1.962
GSK2194060 (nM)
Pictilisib (nM)
Bliss synergy score: 0.598
GW4064 (nM)
PF02545920 (nM)
Bliss synergy score: 3.713
LY−2183240 (nM)
Torcetrapib (nM)
Bliss synergy score: −5.216
MKT 077 (nM)
Bmh−21 (nM)
Bliss synergy score: −1.574
Rosiglitazone (nM)
Dorsomorphin (nM)
Bliss synergy score: −2.965
Rosiglitazone (nM)
Zinterol hydrochloride (nM)

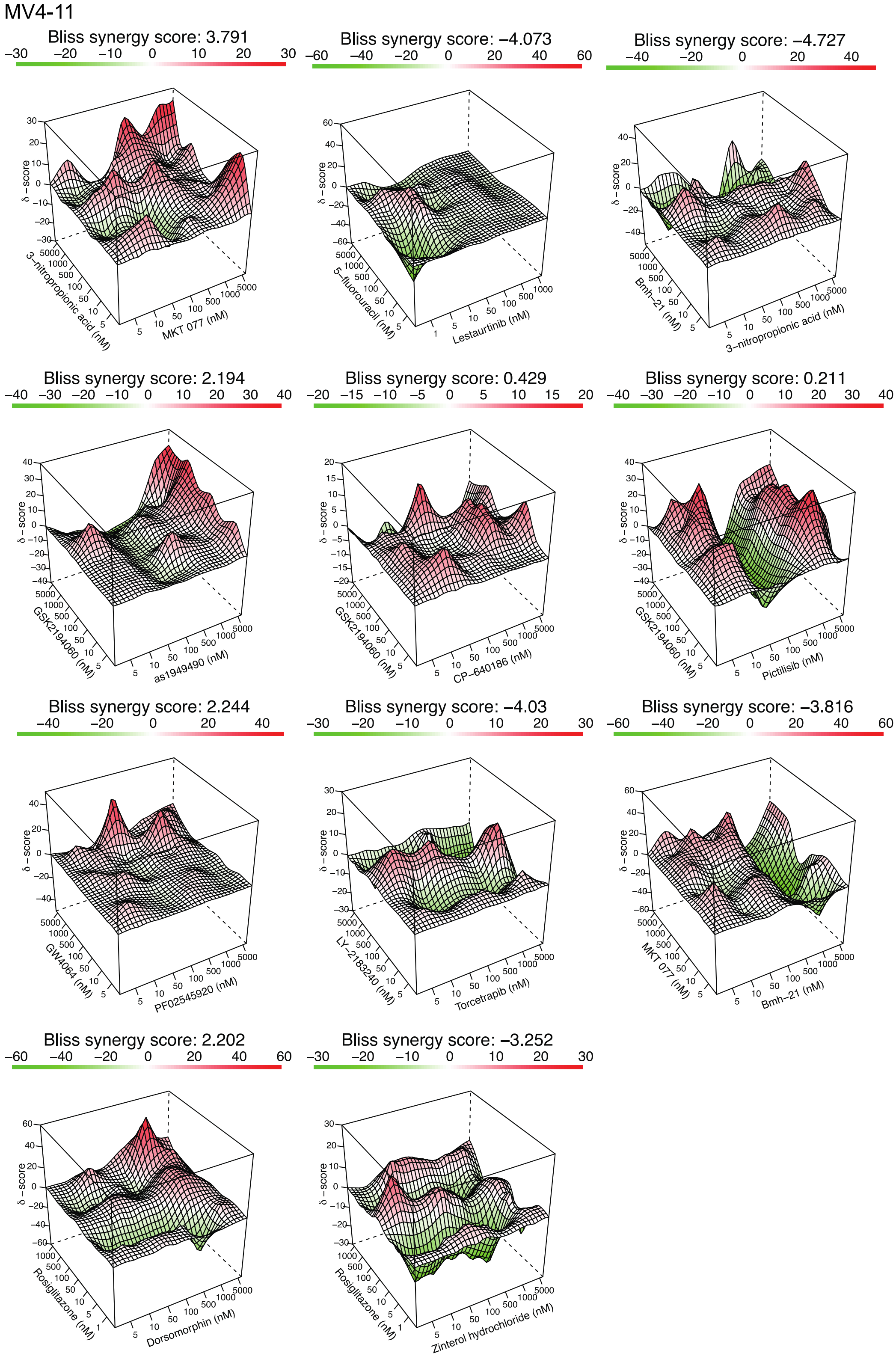

Supplementary Fig. 6| 3D drug synergy visualizations for 11 different drug-drug interactions in HL-60, Mono-Mac-6, BV-173, and MV4-11 cells. Data was generated in 8x8 drug synergy matrices and analyzed using the SynergyFinder tool

using the Bliss independence model with % survival values at each tested concertation relative to DMSO (negative control) and 10μM bortezomib (positive control). The synergy score for a drug combination is averaged over all the dose combination measurments. The 2D and 3D synergy maps highlight synergistic and antagonistic dose regions in red and green, respectively.

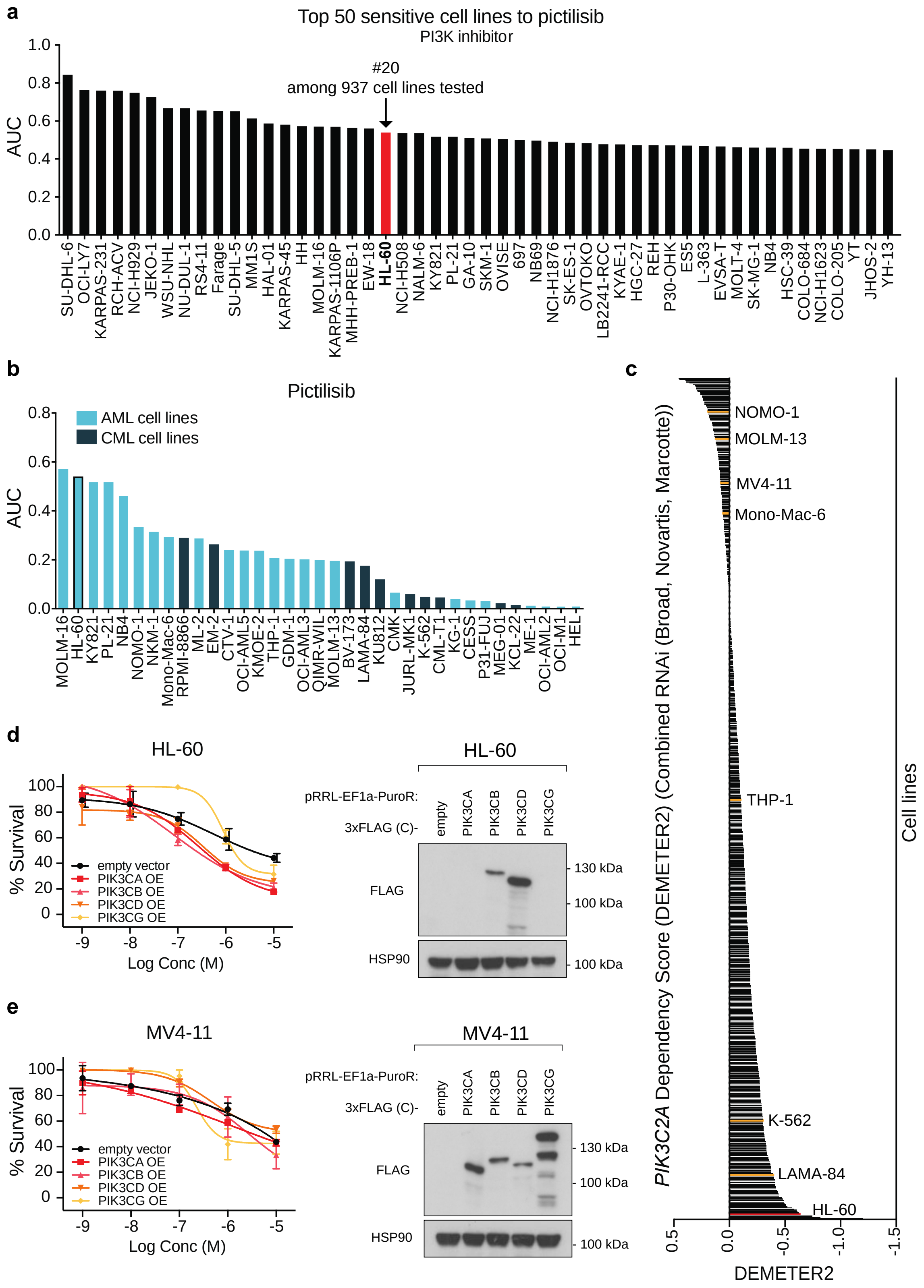
a
Top 50 sensitive cell lines to pictilisib
PI3K inhibitor
#20
among 937 cell lines tested
AUC
b
Pictilisib
AML cell lines
CML cell lines
AUC
c
PIK3C2A Dependency Score (DEMETER2) (Combined RNAi (Broad, Novartis, Marcotte))
Cell lines
DEMETER2
d
HL-60
% Survival
Log Conc (M)
empty vector
PIK3CA OE
PIK3CB OE
PIK3CD OE
PIK3CG OE
pRRL-EF1a-PuroR:
3xFLAG (C)-
empty
PIK3CA
PIK3CB
PIK3CD
PIK3CG
FLAG
HSP90
130 kDa
100 kDa
e
MV4-11

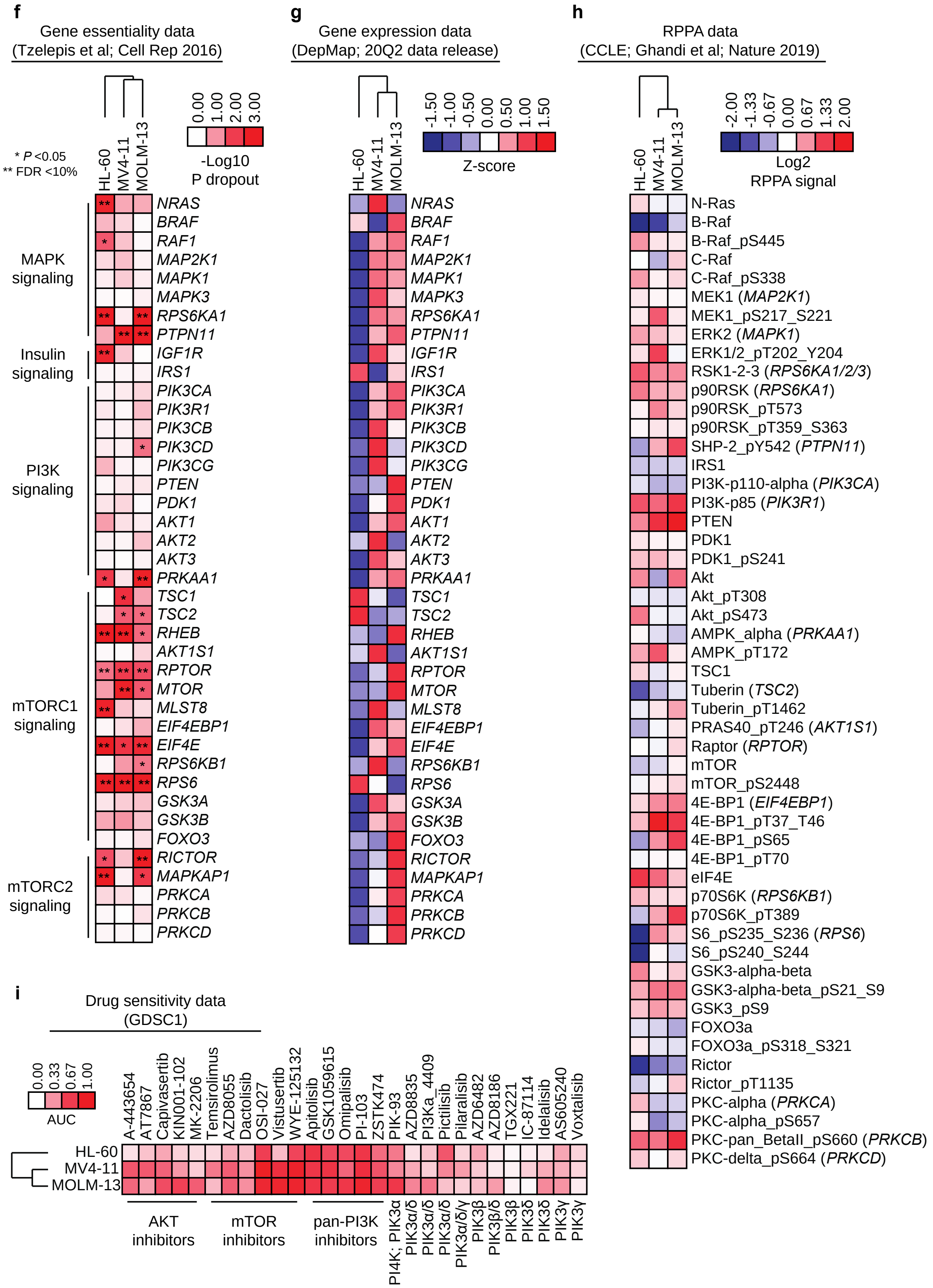

Supplementary Fig. 7| HL-60 cells are vulnerable to PI3K inhibition. a, Distribution of *in vitro* sensitivity (expressed as AUC) to pictilisib among 937 cancer cell lines tested

in the Genomics of Drug Sensitivity in Cancer project (GDSC1)[2]. Data is shown from the top 50 most sensitive lines irrespective of tissue origin or lineage. **b**, Distribution of sensitivity to pictilisib in AML and CML cell lines only. **c**, Cancer cell line genetic dependency to *PIK3C2A* estimated using the DEMETER2 model[3] across 648 cancer cell lines (data retrieved from Cancer Dependency Map portal). The cell lines from this study for which genetic vulnerability data was available are marked. **d**-**e**, Average response to pictilisib in HL-60 and MV4-11 empty vector, and *PIK3CA*, *PIK3CB*, *PIK3CD* or *PIK3CG* overexpressing cells. Error bars indicate standard deviation of the mean of technical triplicates. Data is representative of two independent experiments. Moreover, immunoblot analysis of PIK3CA, PIK3CB, PIK3CD, and PIK3CG expression in HL-60 and MV4-11 cells lentivirally transduced with empty vector, *PIK3CA, PIK3CB, PIK3CD* or *PIK3CG*-cDNA is shown. Data is representative of two independent experiments. **f-l**, Gene essentiality[4], gene expression[5], reverse phase protein array (RPPA)[6], and drug sensitivity data[2], respectively, related to the PI3K/AKT/mTOR signaling in HL-60, MV4-11, and MOLM-13 cells.

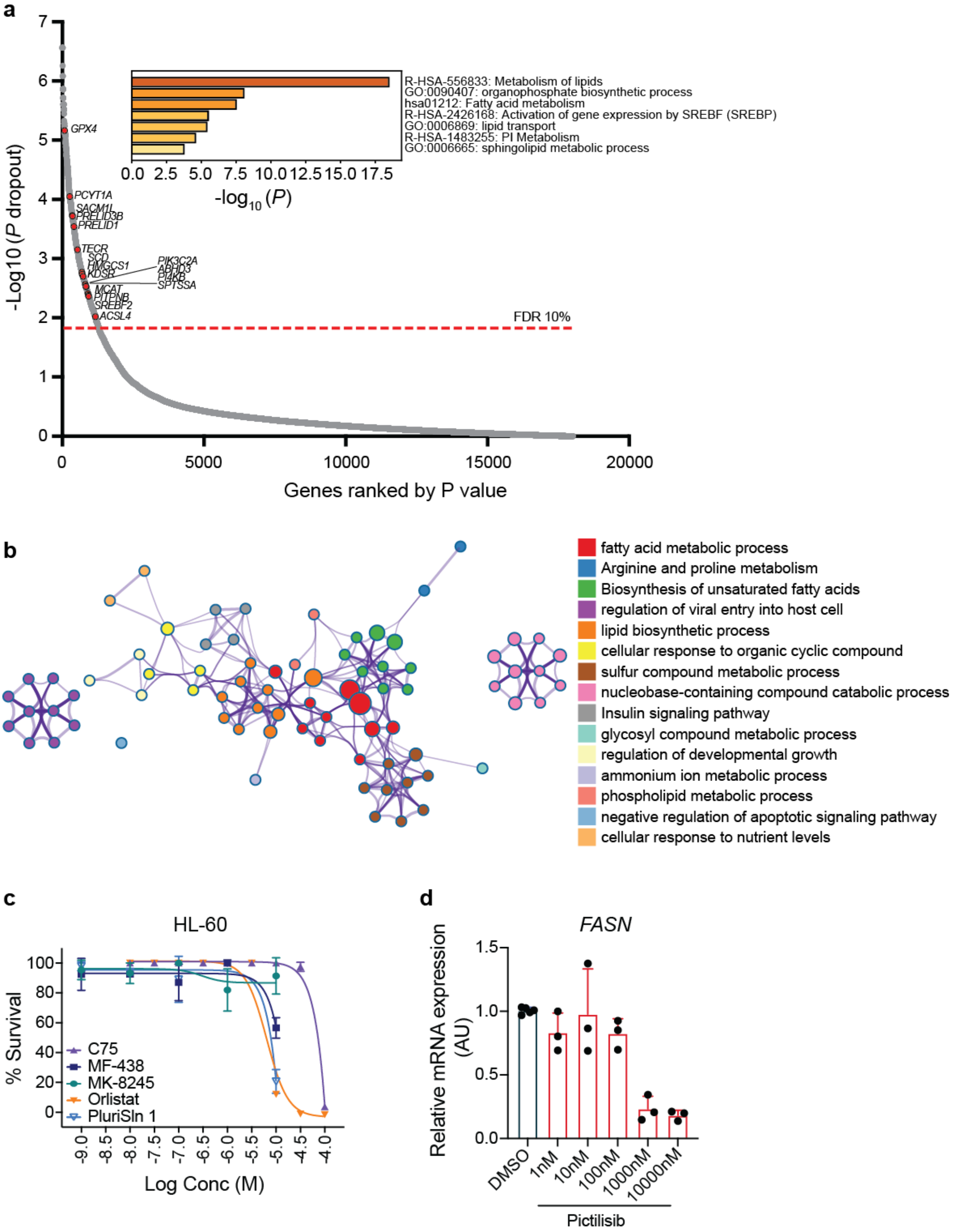

Supplementary Fig. 8| HL-60 cells are dependent on *de novo* FA synthesis. a, Results of genome-wide CRISPR library dropout screen in HL-60 cells. Data obtained from the Yusa lab[4]. Genes involved in lipid metabolism are highlighted in red and gene

enrichment analysis performed with Metascape[7]. **b**, A network layout for enriched ontology clusters of downregulated genes in HL-60 cells generated with Metascape. The network is visualized with Cytoscape (v3.1.2) with “force-directed” layout and with edge bundled for clarity. **c**, Dose response of C75 and orlistat (described to target FASN; technical quadruplicate) as well as 3 stearoyl-CoA desaturase 1 (SCD1; technical duplicate data from original screen) inhibitors in HL-60 cells. Error bars indicate mean ± SD. **d**, *FASN* transcript expression measured by RT-qPCR in response to increasing concentrations of pictilisib treatment. Error bars indicate mean ± SD; (n = 3). Data is representative of two independent experiments

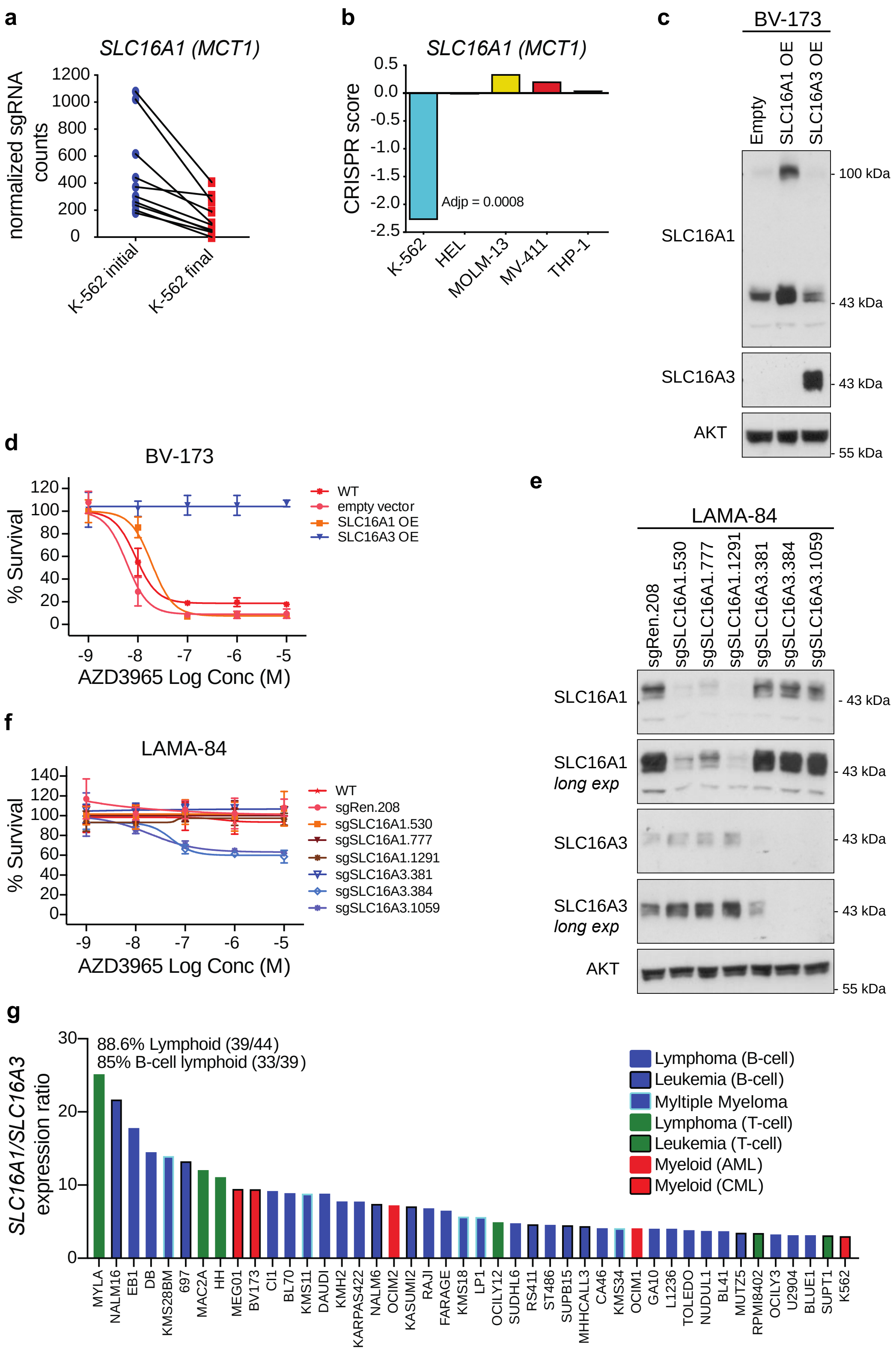

Supplementary Fig. 9| *SLC16A1* essentiality and determinants of sensitivity to the SLC16A1 inhibitor AZD3965. a, Depletion of normalized sgRNA (n = 10) counts for *SLC16A1* in K-562 cells from a CRISPR dropout screen[8], showing that K-562 cells

are dependent on *SLC16A1*. **b**, Comparison of the CRISPR score (a metric to evaluate CRISPR dropout screens and genetic dependencies developed in the Sabatini lab) for the *SLC16A1* in K-562, HEL, MOLM-13, MV4-11, and THP-1 cells. **c**, Immunoblot analysis of SLC16A1 and SLC16A3 expression in BV-173 cells lentivirally transduced with empty vector, *SLC16A1* or *SLC16A3*-cDNA. **d**, Average response to AZD3965 in BV-173 wild type, empty vector, and *SLC16A1* or *SLC16A3* overexpressing cells. **e**, Expression of *SLC16A1* and *SLC16A3* in LAMA-84 CML cells transduced with the indicated sgRNAs. Error bars indicate standard deviation of the mean of technical triplicates. Data is representative of two independent experiments. **f**, Average response to AZD3965 in LAMA-84 wild type, sg*Ren*, and *SLC16A1* or *SLC16A3* deficient cells. Error bars indicate standard deviation of the mean of technical triplicates. Data is representative of two independent experiments. **g**, Distribution of the *SLC16A1*/*SLC16A3* expression ratio across the 44 hematological cancer cell lines predicted to exhibit sensitivity to AZD3965. Bars are colored based on lineage and broad disease subtype. Gene expression data was accessed from the DepMap portal (Expression Public 20Q2)[5].

Supplementary Table 1. Patients' characteristics

**Sample ID**	**Age**	**Sample type**	**Diagnosis**	**Genetics**	**Status at sampling**	**Treatment before sampling**
Pat.1	> 65	BM	AML with MDS-related changes	No mutations detected	Diagnostic	
Pat.2	< 65	BM	CML, chronic phase	BCR-ABL	Diagnostic	
Pat.3	< 65	PB	CML, blast crisis	BCR-ABL	Refractory disease	dasatinib<br>imatinib<br>nilotinib<br>bosutinib<br>FLAG<br>ponatinib
Pat.4	> 65	PB	AML NOS	*NRAS* G12D	Refractory disease	3+5+7 (daunorubicin, cytarabine, etoposide) followed by mitoxantrone, cytarabine
Pat.5	> 65	PB	sAML post PV	*FLT3*-ITD<br>*JAK2* V617F	Diagnostic	
Pat.6	< 65	PB	AML with mutant NPM1	*NPM1*-B<br>*TP53* polymorphism R72P	Relapse	high dose cytarabine
Pat.7	< 65	BM	CML, blast crisis	BCR-ABL_T315I	Relapse	imatinib<br>nilotinib<br>dasatinib
Pat.8	> 65	BM	AML with mutant NPM1	*NPM1*-A	Diagnostic	
Pat.9	< 65	PB	AML with mutant NPM1	*NPM1*-D<br>*FLT3*-ITD	Relapse	fludarabine<br>cyclophosphamide<br>allogeneic cord blood transplant

Pat.10	> 65	PB	sAML post MPN	*CALR* L367fs*46	Relapse	azacytidine<br>venetoclax
Pat.11	< 65	PB	AML with MDS-related changes	*TP53* R282W heterozygous<br>*TP53* R72P	Relapse	allogenous stem cell transplant<br>cyclophosphamide<br>methotrexate<br>total body irradiation
Pat.12	< 65	PB	AML	N/A	Relapse	azacytidine
Pat.13	> 65	PB	sAML post MDS	DEK-NUP214<br>*FLT3*-ITD	Relapse	azacytidine<br>hydroxyurea
Pat.14	> 65	PB	AML with MDS-related changes	*IDH1* R132C<br>*RUNX1* F40Wfs*14<br>*RUNX1* R162K<br>*CBL* C404Y	Relapse	azacytidine
Pat.15	> 65	BM	sAML post MDS/MPN unclassifiable	*ASXL1* Q512*<br>*FLT3*-ITD<br>*PTPN11* S506T<br>*SETBP1* G870S<br>*SRSF2* P95R	Relapse	venetoclax<br>decitabine

MDS-myelodysplastic syndrome; FLAG-fludarabine, arabinofuranosyl cytidine, and granulocyte colony-stimulating factor; NOS-not otherwise specified; sAML-secondary AML; PV-polycythemia vera; MPN-myeloproliferative neoplasm; N/A-not available.

Supplementary References

1 Li, H. *et al.* The landscape of cancer cell line metabolism. *Nat Med* **25**, 850-860, doi:10.1038/s41591-019-0404-8 (2019).

2 Yang, W. *et al.* Genomics of Drug Sensitivity in Cancer (GDSC): a resource for therapeutic biomarker discovery in cancer cells. *Nucleic Acids Res* **41**, D955-961, doi:10.1093/nar/gks1111 (2013).

3 McFarland, J. M. *et al.* Improved estimation of cancer dependencies from large-scale RNAi screens using model-based normalization and data integration. *Nat Commun* **9**, 4610, doi:10.1038/s41467-018-06916-5 (2018).

4 Tzelepis, K. *et al.* A CRISPR Dropout Screen Identifies Genetic Vulnerabilities and Therapeutic Targets in Acute Myeloid Leukemia. *Cell Rep* **17**, 1193-1205, doi:10.1016/j.celrep.2016.09.079 (2016).

5 DepMap, B. DepMap 20Q2 Public. figshare. Dataset. https://doi.org/10.6084/m9.figshare.12280541.v4. (2020).

6 Ghandi, M. *et al.* Next-generation characterization of the Cancer Cell Line Encyclopedia. *Nature* **569**, 503-508, doi:10.1038/s41586-019-1186-3 (2019).

7 Zhou, Y. *et al.* Metascape provides a biologist-oriented resource for the analysis of systems-level datasets. *Nat Commun* **10**, 1523, doi:10.1038/s41467-019-09234-6 (2019).

8 Wang, T. *et al.* Identification and characterization of essential genes in the human genome. *Science* **350**, 1096-1101, doi:10.1126/science.aac7041 (2015).
