# Supplementary material for: Metabolic drug survey highlights cancer cell dependencies and vulnerabilities
Source: Nat Commun. 2021 Dec 14;12:7190. doi: 10.1038/s41467-021-27329-x (PMC8671470; doi:10.1038/s41467-021-27329-x)
Supplement: Supplementary file 3 — Description of Additional Supplementary Files [file 41467_2021_27329_MOESM3_ESM.pdf]

Description of Additional Supplementary Files

Supplementary Data 1: CeMM Library of Metabolic Drugs (CLIMET).

Supplementary Data 2: Drug sensitivity profiles of AML and CML cell lines. This supplementary data contains the drug sensitivity data including mean % Survival for each drug tested in each cell line, IC50 values in uM and AUC values used for downstream analysis.

Supplementary Data 3: Drug sensitivity profiles of AML and CML patient samples. This supplementary data contains the drug sensitivity data including mean % Survival for each drug tested in each patient sample and AUC values used for downstream analysis.
