# Supplementary material for: Metabolic drug survey highlights cancer cell dependencies and vulnerabilities
Source: Nat Commun. 2021 Dec 14;12:7190. doi: 10.1038/s41467-021-27329-x (PMC8671470; doi:10.1038/s41467-021-27329-x)
Supplement: Supplementary file 7 — Reporting summary [file 41467_2021_27329_MOESM7_ESM.pdf]

nature research

Corresponding author(s): Giulio Superti-Furga

Last updated by author(s): Nov 2 2021

Reporting Summary

Nature Research wishes to improve the reproducibility of the work that we publish. This form provides structure for consistency and transparency in reporting. For further information on Nature Research policies, see our Editorial Policies and the Editorial Policy Checklist.

Statistics

For all statistical analyses, confirm that the following items are present in the figure legend, table legend, main text, or Methods section.

n/a	Confirmed	
☐	☒	The exact sample size (*n*) for each experimental group/condition, given as a discrete number and unit of measurement
☐	☒	A statement on whether measurements were taken from distinct samples or whether the same sample was measured repeatedly
☐	☒	The statistical test(s) used AND whether they are one- or two-sided<br>*Only common tests should be described solely by name; describe more complex techniques in the Methods section.*
☒	☐	A description of all covariates tested
☐	☒	A description of any assumptions or corrections, such as tests of normality and adjustment for multiple comparisons
☐	☒	A full description of the statistical parameters including central tendency (e.g. means) or other basic estimates (e.g. regression coefficient) AND variation (e.g. standard deviation) or associated estimates of uncertainty (e.g. confidence intervals)
☐	☒	For null hypothesis testing, the test statistic (e.g. *F*, *t*, *r*) with confidence intervals, effect sizes, degrees of freedom and *P* value noted<br>*Give P values as exact values whenever suitable.*
☒	☐	For Bayesian analysis, information on the choice of priors and Markov chain Monte Carlo settings
☒	☐	For hierarchical and complex designs, identification of the appropriate level for tests and full reporting of outcomes
☒	☐	Estimates of effect sizes (e.g. Cohen's *d*, Pearson's *r*), indicating how they were calculated

Our web collection on statistics for biologists contains articles on many of the points above.

Software and code

Policy information about availability of computer code

Data collection	Information on metabolic enzymes and drug target annotations were collected from publicly available databases such as:<br>KEGG https://www.genome.jp/kegg/<br>SMPDB https://smpdb.ca<br>ChEMBL https://www.ebi.ac.uk/chembl/<br>PubChem https://pubchem.ncbi.nlm.nih.gov<br>DrugBank https://www.drugbank.ca<br>Drug approval status was manually curated from product portfolio data of pharmaceutical companies' websites, clinicaltrials.gov and product-specific press releases.<br>CTG viability data were collected on a EnVision multi-label plate reader (PerkinElmer) or a SpectraMax i3x Multi-mode Microplate Reader (Molecular Devices).<br>Mitochondrial respiration and stress data were collected on the XF96 Extracellular Flux Analyzer (Seahorse Biosciences).<br>Western blots were acquired with the ECL Western blotting system (Thermo Fisher Scientific).
Data analysis	Statistical analysis were performed in GraphPadPrism software (v. 8.0- v. 9.0) and R (v. 3.6.0- v. 3.6.2)<br>Software and R packages used in our analysis are listed below:<br>drc R package (v. 3.0-1) https://cran.r-project.org/web/packages/drc/drc.pdf (Ritz, C. PLoS One 2015).<br>PharmacoGx R package (v. 2.6.0) https://www.bioconductor.org/packages/release/bioc/html/PharmacoGx.html (Smirnov, P. et al. Bioinformatics 2016).<br>Pvclust R-package (2.2-0) https://CRAN.R-project.org/package=pvclust (Suzuki, R. & Shimodaira, H. Bioinformatics 2006).

Gene Cluster 3.0 software http://bonsai.hgc.jp/~mdehoon/software/cluster/software.htm
Java Tree View software v. 1.1.6r4 http://jtreeview.sourceforge.net
Combinatorial effects were evaluated and visualized using the SynergyFinder 2.0 web portal (https://synergyfinder.fimm.fi) using the Bliss independent model.
MetaboAnalyst 3.0 https://www.metaboanalyst.ca (Chong, J. et al. Curr Protoc Bioinformatics 2019; Chong, J. et al. Metabolites 2019).
Corplot R package (v. 0.90) (Wei, T. & Simko, V. R package "corrplot": Visualization of a Correlation Matrix (Version 0.84). 2017)
Metascape https://metascape.org/gp/index.html#/main/step1 (Zhou, Y. et al. Nat Commun 2019)
Gene Ontology Resource http://geneontology.org (Ashburner, M. et al. Nat Genet 2000; The Gene Ontology, C. Nucleic Acids Res 2019).
CHOPCHOP https://chopchop.cbu.uib.no (Montague, T. G. et al. Nucleic Acids Res 2014).
Venn webtool from the University of Gent (http://bioinformatics.psb.ugent.be/webtools/Venn/)

For manuscripts utilizing custom algorithms or software that are central to the research but not yet described in published literature, software must be made available to editors and reviewers. We strongly encourage code deposition in a community repository (e.g. GitHub). See the Nature Research guidelines for submitting code & software for further information.

Data

Policy information about availability of data

All manuscripts must include a data availability statement. This statement should provide the following information, where applicable:

- Accession codes, unique identifiers, or web links for publicly available datasets
- A list of figures that have associated raw data
- A description of any restrictions on data availability

Information on metabolic enzymes and drug target annotations are available from publicly accessible databases such as KEGG (https://www.genome.jp/kegg/); SMPDB (https://smpdb.ca); ChEMBL (https://www.ebi.ac.uk/chembl/), PubChem (https://pubchem.ncbi.nlm.nih.gov), and DrugBank (https://www.drugbank.ca). The manually curated CLIMET compound annotations are provided as Supplementary Data 1. Drug screening data generated in this study are provided as Supplementary Data 2 and 3. The Genomics of Drug Sensitivity in Cancer publicly available drug sensitivity data used in this study are available on the GDS portal (https://www.cancerrxgene.org; GDSC1 data release). The DepMap cell line annotations (DepMap Public 20Q2; sample_info.csv), CCLE cell line targeted metabolomics (CCLE_metabolomics_20190502.csv; expressed as Log10-transformed values cleaned up data), somatic mutation (DepMap 19Q3 Public. figshare. Dataset. https://doi.org/10.6084/m9.figshare.9201770.v1. (2019).), gene expression (DepMap 19Q3 Public. figshare. Dataset. https://doi.org/10.6084/m9.figshare.9201770.v1. (2019); DepMap 20Q2 Public. figshare. Dataset. https://doi.org/10.6084/m9.figshare.12280541.v4. (2020)) expressed as Log2 (TPM+1)), RNAi (D2_combined_gene_dep_scores.csv), and RPPA (CCLE_RPPA_20181003.csv expressed as Log2 RPPA signal) data used in this study are available from the Broad DepMap portal (https://depmap.org/portal/download/). Other gene dependency datasets used are available from the Supplementary Information of the original publications (Tzelepis, K. et al. Cell Rep 2016, Wang, T. et al. Science 2015, and Wang, T. et al. Cell 2017). Patient sample gene expression data from The Cancer Genome Atlas (TCGA) can be accessed via the UCSF Xena project (http://xena.ucsc.edu) visualized with the GEPIA server (http://gepia.cancer-pku.cn/index.html). AML patient sample drug sensitivity data used in this study is available at http://www.vizome.org/aml/. Complete immunoblot scans are available in the source data file. All other data generated in this study and/or supporting the findings of this study is available within the paper, its supplementary information files and the source data accompanying the paper. Source data are provided with this paper.

Field-specific reporting

Please select the one below that is the best fit for your research. If you are not sure, read the appropriate sections before making your selection.

☒ Life sciences ☐ Behavioural & social sciences ☐ Ecological, evolutionary & environmental sciences

For a reference copy of the document with all sections, see nature.com/documents/nr-reporting-summary-flat.pdf

Life sciences study design

All studies must disclose on these points even when the disclosure is negative.

Sample size	Sample size was determined empirically, based on exploratory experiments, our previously published work as well as published literature with similar methodology. No statistical test was performed to predetermine sample size. For the publicly accessible data used in this study all cell lines and or patient samples of relevance available were used (e.g. data on SLC16A1 and SLC16A3 gene expression in all hematological malignancy cell lines available in the Cancer Dependency Portal and in patients samples obtainable through The Cancer Genome Atlas). All experiments were repeated as indicated in methods and in the figure legends.
Data exclusions	Compounds that did not affect cell viability at any of the tested drug concentrations were excluded from downstream analysis. Genes that were not expressed in any of the cell lines of interest (expression level Log2 (TPM+1)<1) were excluded from downstream analysis. These were pre-established exclusion criteria standardly applied to these data types and did not affect any experiments or downstream analysis.
Replication	For the initial drug screening of the metabolic drug library (CLIMET) each drug was tested in 5 different concentrations in a technical duplicate. Validation experiments for selected compounds were performed in at least 2 biological replicates with 3 technical replicates each. All attempts at replication were successful.
Randomization	The samples for each experiment were randomized to be examined (No specific methods were used).

Blinding	No blinding was performed due to none of the analyses reported involved procedures that could be influenced by investigator bias.

Reporting for specific materials, systems and methods

We require information from authors about some types of materials, experimental systems and methods used in many studies. Here, indicate whether each material, system or method listed is relevant to your study. If you are not sure if a list item applies to your research, read the appropriate section before selecting a response.

Materials & experimental systems

n/a	Involved in the study
☐	☒ Antibodies
☐	☒ Eukaryotic cell lines
☒	☐ Palaeontology and archaeology
☒	☐ Animals and other organisms
☐	☒ Human research participants
☒	☐ Clinical data
☒	☐ Dual use research of concern

Methods

n/a	Involved in the study
☒	☐ ChIP-seq
☒	☐ Flow cytometry
☒	☐ MRI-based neuroimaging

Antibodies

Antibodies used	a-SLC16A1 (Santa Cruz Biotechnology; sc-365501), a-SLC16A3 (Santa Cruz Biotechnology; sc-376140), a-AKT (Cell Signaling Technologies; #4685), a-FLAG (F1804, Sigma-Aldrich), a-HSP90 (610418, BD Biosciences), Peroxidase AffiniPure Goat Anti-Mouse IgG (Jackson ImmunoResearch; 115-035-003), Peroxidase AffiniPure Goat Anti-Rabbit IgG (Jackson ImmunoResearch; 111-035-003).
Validation	All antibodies purchased were validated by their manufacturer for the appropriate application on cells expressing the antigen of interest and further information is available on the manufacturers website or the provided scientific citations on the same website. Moreover, the antibodies used were validated by their respective molecular weight. Specificity of SLC16A1 and SLC16A3 antibodies were further validated by western blot in SLC16A1/SLC16A3 knockout and overexpressing cells (see for example Fig. 6c and e, Supplementary Figure 8c and e). Akt (pan) (11E7) rabbit mAb detects endogenous levels of total Akt protein. Western blot staining has been validated on recombinant Akt1, Akt2 and Akt3 proteins, and extracts from HeLa, C2C12, C6 and COS cells (data available on the manufacturer's website). The ANTI-FLAG M2 monoclonal antibody detects epitope-tagged fusion proteins by common immunological procedures such as Western blotting with staining being validated in FLAG protein spiked CHO cells lysate (https://www.sigmaaldrich.com/deepweb/assets/sigmaaldrich/product/documents/144/194/vol6_iss2_antiflag_m2.pdf). The purified mouse-anti-Hsp90 antibody recognizes endogenous levels of human Hsp90 protein and its specificity has been validated in HeLa cell lysates with increasing concentration of antibody (data available on the manufacturer's website). Moreover, these antibodies have been previously used in another study from our laboratory (Bigenzahn et al; Science 2018).

Eukaryotic cell lines

Policy information about cell lines

Cell line source(s)	MOLM-13, MV4-11, Mono-Mac-6, KG-1, SHI-1, ML-2, NOMO-1, HEL, LAMA-84, KU-812, KCL-22, and BV-173 were obtained from DSMZ, K-562 and HL-60 from NCI 60 panel and THP-1 from ATCC
Authentication	All cell lines used in this study were authenticated by STR profiling.
Mycoplasma contamination	Cell lines used were tested negatively for mycoplasma contamination.
Commonly misidentified lines (See ICLAC register)	None of commonly misidentified cell lines were used in this study.

Human research participants

Policy information about studies involving human research participants

Population characteristics	The relevant population characteristics can be found in Supplementary Table 3 of the manuscript.
Recruitment	AML and CML patients were not specifically recruited, but only available viably frozen samples from diagnostic or refractory AML and CML patient samples were used as a comparison cohort to the originally tested cell line panel.
Ethics oversight	Ethical approval was granted by the Ethics Commission of the Medical University of Vienna (Ethik Kommission 1676/2016).

Note that full information on the approval of the study protocol must also be provided in the manuscript.
